# Annotations

**Published:** 1908-01-11

**Authors:** 


### ANNOTATIONS.

### The Church Bell Nuisance.

Not a few people in this country will long for the possession of municipal powers similar to those under which, according to a recent report, the Mayor of a French Commune has forbidden the local curé to ring the church bell for more than three minutes a day. Indeed, an even more complete prohibition would not be undesirable. The church bell has, of course, its poetical and sentimental associations, and at one time it doubtless had its practical value. But in this age of cheap clocks and watches it is no longer necessary, and for those who live in its immediate neighbourhood it is oft-times a considerable nuisance. It remains, however, as an example of the essential conservatism of the British people, and we can hardly hope that our protest here recorded will silence it. Possibly, indeed, this comment will excite anything but sympathy, and some of our readers may be inclined actively to condemn our attitude on the question. Yet we are quite sure that were any institutions other than those of ecclesiastical function to set up a similar method of announcing to the world the times of their activity, there would be a furious and unanimous popular outcry. What justifies the introduction of the subject in these columns is the disastrous effect produced by the ringing of church bells upon the comfort and welfare of many invalids. Every medical practitioner is familiar with instances that fall within this category, and occasionally the experience is a very painful one. A sleep that would be most beneficial, that might even possibly turn the issue between life and death, is prevented or rudely broken by the fierce clanging which issues from a neighbouring steeple, or by, what is not uncommon, the ear-splitting discords of neighbouring steeples. If the nuisance cannot be altogether suppressed, it should at least be temporarily suspended on the veto of any one living in the neighbourhood. There are many unnecessary noises, and the church bell is one of them.

### Science and the Public.

A somewhat amusing correspondence under this title has recently been in progress in the *Times*, and in the course of it there has been some pretty plain speaking both on one side and the other. The original proposition was that the complaint of many scientific men that the public takes but an indifferent interest in their work is no doubt true, but the fault, it was argued, lies with the scientists themselves. When these distinguished men come before the public, as they do in various courses of popular lectures, they show—such is the accusation—that they do not know their business as teachers—"they fumble their laboratory notes before the unfortunate audience and call them a lecture." As opposed to this a learned Professor urges that both by education and by inclination the general public is unfitted to profit by the popular appearances of himself and his colleagues. They take no interest in scientific subjects nor in the application of science to the world of practical affairs, and the questions addressed to the lecturer at the close of his discourse frequently show that the questioners are no wiser at the end of the lecture than they were at the beginning. It is difficult to reconcile the first of these charges with the crowded audiences which many popular science courses command, and if it is true that pupils receive no benefit it is at least possible that the fault lies not with them but with the teacher. The complaints here advanced against popular audiences are very similar to those heard in some of our medical schools. Teachers who fail to teach must find some excuse, and the stupidity of pupils naturally occurs to them as a convenient explanation. The fact is that in scientific circles teaching ability is altogether undervalued. The younger men find it receives but little encouragement in the struggle for promotion, and some of them learn from the example of their seniors that popular success of this order is a thing to be despised rather than encouraged.

### Medical Men and Death Certificates.

Dr. Henry Daniell, of Nassau Road, Barnes, was fined for refusing to give a death certificate in the case of triplets, who died within an hour of birth. The doctor had, indeed, written some lines on a sheet of paper, but it was so unsatisfactory that the cemetery authorities would not consent to the burial of the infants on the strength of it. In the evidence it appeared that the mother had been prematurely confined of triplets, all of whom died, but although the doctor declared that the nurse had done her duty perfectly, and that he had no suspicions in regard to the parents, he refused to give a certificate as to the cause of death, and said that he was not quite clear as to the reason of the premature birth, and "desired to shelter himself behind the coroner." On the face of it this seems rather a pusillanimous resolve, and so far as one can judge from the printed reports there was nothing to justify Dr. Daniell's timidity. The Medical Officer of Health, who examined the bodies of the three infants, found no reason to suspect any improper cause for the premature birth. Indeed, the mere fact of there being three infants would be, to the ordinary mind, a sufficient explanation of such an accident. Only exceptional women can bear three children to the full time. But Dr. Daniell might have escaped unfavourable criticism if he had not asked, when a certificate was demanded, "Who is going to pay me?" and charged a Miss Pincham 2s. for the paper on which he certified the death without stating a cause. Whatever justification Dr. Daniell might have had for his action was greatly weakened by this question as to fees. If he had doubts as to whether the premature birth had been wilfully brought on, he should have refused a certificate and communicated with the coroner; if not, the certificate should have been given, and he should have taken the ordinary means to recover the proper fee due to him. It is quite possible that his conduct was wholly upright, but by asking who was to pay him, and by saying that his indefinite certificate was given because he wanted to shelter himself behind the coroner, he put himself in the wrong, and one cannot regret that the result of the inquiry was that he was compelled to pay a fine of 40s. and costs in the case of one child, and the costs of the other cases. If a doctor cannot give a straight opinion in such cases, whom can we expect to do so?

